# Linking Reactive Oxygen Species (ROS) to Abiotic and Biotic Feedbacks in Plant Microbiomes: The Dose Makes the Poison

**DOI:** 10.3390/ijms23084402

**Published:** 2022-04-15

**Authors:** Louis Berrios, Jeremy D. Rentsch

**Affiliations:** 1 Department of Biology, Stanford University, Stanford, CA 94305, USA; 2 Department of Biology, Francis Marion University, Florence, SC 29502, USA; jrentsch@fmarion.edu

**Keywords:** abiotic–biotic feedbacks, reactive oxygen species (ROS), plant microbiome

## Abstract

In nature, plants develop in complex, adaptive environments. Plants must therefore respond efficiently to environmental stressors to maintain homeostasis and enhance their fitness. Although many coordinated processes remain integral for achieving homeostasis and driving plant development, reactive oxygen species (ROS) function as critical, fast-acting orchestrators that link abiotic and biotic responses to plant homeostasis and development. In addition to the suite of enzymatic and non-enzymatic ROS processing pathways that plants possess, they also rely on their microbiota to buffer and maintain the oxidative window needed to balance anabolic and catabolic processes. Strong evidence has been communicated recently that links ROS regulation to the aggregated function(s) of commensal microbiota and plant-growth-promoting microbes. To date, many reports have put forth insightful syntheses that either detail ROS regulation across plant development (independent of plant microbiota) or examine abiotic–biotic feedbacks in plant microbiomes (independent of clear emphases on ROS regulation). Here we provide a novel synthesis that incorporates recent findings regarding ROS and plant development in the context of both microbiota regulation and plant-associated microbes. Specifically, we discuss various roles of ROS across plant development to strengthen the links between plant microbiome functioning and ROS regulation for both basic and applied research aims.

## 1. Introduction

Photosynthesis has evolved fundamental and dual roles for reactive oxygen species (ROS) [1]. ROS—which include singlet oxygen (^1^O_2_), superoxide (O^2•−^), peroxide ion (O_2_^2−^), hydrogen peroxide (H_2_O_2_), and hydroxyl radical (•OH)—are derived from oxygen (O_2_) and drive diverse cellular and organismal outcomes that range from cell proliferation and immune system responsiveness to cellular death and senescence [2,3,4]. Thus, ROS facilitate plant growth and development. Whether ROS function in regulatory or lethal capacities depends on their local and systemic concentration [5] as well as their timing and location of production [6]. A plant’s enzymatic capacity (i.e., the plant processing system) largely determines its ‘homeostatic potential’—defined here as the capacity to regulate cellular and organismal stasis. However, the ephemeral nature of some ROS molecules also leads to unique, molecule-specific features that further shape homeostatic potentials (see Mhamdi and Van Breusegem 2018 for a detailed review [7]. Nevertheless, reports have linked ROS to the successful disruption of seed dormancy and the subsequent initiation of seedling-to-plant development [8], and the concentrations of unique ROS forms (e.g., H_2_O_2_ versus O^2•−^) have been mapped to the location of developing root apical meristems [9]. In contrast, high or deleterious concentrations of ROS (i.e., oxidative stress) can cause mutagenic DNA strand breaks, purine oxidations, and protein–DNA cross links, which can result in organismal senescence [10,11]. Therefore, the spatiotemporal balance of ROS concentrations dictates whether ROS facilitate or diminish plant growth and development.

**Figure 1:**
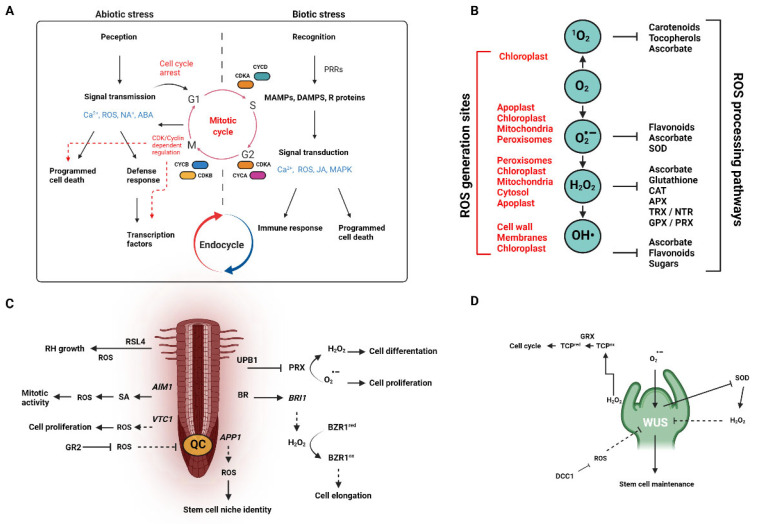
ROS as they generally relate to the cellular and organismal processes of plant growth and development. (**A**) Schematic of abiotic and biotic signaling processes in relation to plant cell cycle regulation (adapted from [39]). (**B**) General overview of ROS, their generation sites, and the ROS processing pathways (both enzymatic and non-enzymatic; adapted from [7]. (**C**) Root apical meristem (RAM) development and shoot apical meristem (**D**) (SAM) development (see [4,40] for a detailed overview) as and the signaling role(s) played by selected ROS types.

**Figure 2:**
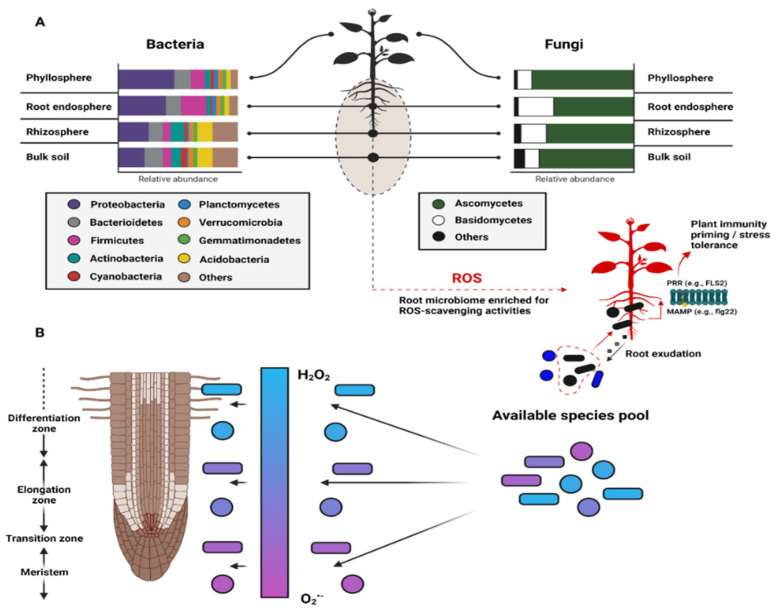
Plant microbiome composition and shifts as a function of ROS. (**A**) Bacterial and fungal taxa relative abundances in the bulk soil, rhizosphere, root endosphere, and phyllosphere, where plants selectively enrich for microbes with efficient ROS scavenging abilities (**B**) across a predictable ROS gradient. Following this conceptual model, we could expect to observe distinct taxa and/or functional complements that compartmentalize as a function of the niche conditions. Clarifying how these spatiotemporal shifts affect the spatiotemporal dynamics of plant development remains critical for advancing the field of plant–microbe interactions and microbial ecology.

Coupled to the dual roles that ROS play in plant development lies the occurrence of interacting abiotic and biotic factors innate to complex, adaptive systems. In nature, plants encounter both abiotic and biotic stresses that sum to shape their proximate and ultimate fates. Given their sessile status, plants thus rely, in part, on their microbial counterparts (collectively termed the plant microbiome) to buffer the effects of environmental stress(es) [12]. During these stressed states, the plant microbiome can facilitate plant homeostasis [13] via direct and/or indirect mechanisms (see Trivedi et al. 2020 for a comprehensive review [14]). Though the way(s) in which plant microbiomes reconfigure to support host homeostasis remain(s) an active field of research, several factors surrounding the interplay between plants and their microbiota have been reported over the last few decades [15,16,17], and strong evidence suggests that ROS facilitate abiotic–biotic feedbacks [18] (see Figure 1 and Figure 2 for cartoon schematics). For example, a recent suite of analyses demonstrated that plants selectively limit the proliferation of select bacterial plant microbiome members through reactive oxygen species mechanisms (see Stringlis et al. 2021 for a recent review [19], while others have reported the genomes of symbiotic bacteria tend to be enriched in ROS scavenging encoding genes [20]. Moreover, several microbes have been shown to enhance plant fitness by reducing deleterious ROS levels in various plant compartments (see Nath et al. 2016 and Singh et al. 2021 for comprehensive reviews [21,22]). However, clear mechanisms that link the community ecology of plant microbiota in the context of ROS have not yet been communicated [23]. Specifically, the degree to which plants selectively filter out microbial taxa incapable of living in high oxidative stress conditions (e.g., proximal or inside the developing root of a stressed plant) or select for ROS-scavenging microbes and/or those that provide additional benefit(s) to their plant partner(s) (e.g., facilitating nutrient uptake or providing key phytohormones) remains unclear [24]. Clarifying the feedbacks that drive the homeostasis of plants and their microbiota could facilitate tremendous insights across agroecological systems.

In this review, we summarize the developmental function(s) of ROS in plants and outline the involvement of ROS as they relate to abiotic–biotic feedbacks in plant microbiomes. Comprehensive reviews on the topics of ROS signaling in plants [7] and abiotic–biotic feedbacks in plant microbiomes [25] have already been communicated. Here we aim to synthesize novel insights that home in on the regulatory and stimulatory effects of plant microbiota through the scope of ROS homeostasis. To this end, we frame much of our discussion around strong interactions between plants and microbes that relate to host–microbe recognition [26,27,28,29,30], kingdom-directed plant–microbe interactions [31], priority effects (i.e., when species arrive to a given environment) in plant microbiomes [32], and agroecological relevance [33,34].

## 2. ROS and Plant Homeostasis

### 2.1. Seed Germination and Root Development

There is now overwhelming evidence that ROS play critical roles in the regulation of plant growth and development from germination to senescence (Figure 1A). Germination is a complex process by which desiccated seeds rehydrate, which then triggers a cascade of metabolic events that lead to the emergence of the seedling [35]. Along with these metabolic changes come a reorganization of cellular structures, the activation of protective systems, and the loss of desiccation tolerance [36]. Sharp increases in both superoxide anion (O_2_^·−^) and hydrogen peroxide (H_2_O_2_) have been detected reliably in the transition from dormancy to germination in *Helianthus annuus* L. seeds through the invocation of specific patterns of carbonylation [37]. However, in the case where cytosolic APX6, a peroxide-scavenging enzyme, is knocked out, germination rates decrease due to increased oxidative damage [38]. This suggests that the precise pattern of carbonylation is important. These results also confirm the now well-established doctrine that both the synthesis and scavenging of ROS are important for maintaining homeostasis and that ROS homeostasis is important in the utilization of these volatile molecules as signaling molecules (see Figure 1B). 

Radical elongation and endosperm weakening (prior to endosperm rupture) co-occur with an increase in ROS levels (Figure 1C). The treatment of pea (*Pisum sativum* L.) seeds with H_2_O_2_ has been shown to increase both germination percentages and growth rates [41]. This general result has been seen in other taxa, ranging from *Cinnamomum camphora* (L.) J. Presl. [42] to *Zinnia elegans Jacq* [43]. Similarly, in barley (*Hordeum vulgare* L.) H_2_O_2_ is required to break dormancy—most likely by the induction of HvGA20ox1, which is involved in gibberellic acid (GA) synthesis. It is of interest to note here that H_2_O_2_ treatment also leads to slightly elevated abscisic acid (ABA) levels, re-enforcing the idea that breaking seed dormancy is a matter of ABA/GA balance and not simply the complete dominance of one hormone over another [44]. It should also be noted that this is an evolving field and much of the nuance is likely to be lineage specific. For example, in *Bidens pilosa* L., H_2_O_2_ does not appear to facilitate the breaking of seed dormancy—although other reactive species of oxygen (•OH and O^2•−^) evidently do [45]. On the contrary, in *Castanea sativa Mill*., extracellular O^2•−^ production preceded desiccation-induced viability loss [46], perhaps owing to the recalcitrant nature of the seeds. GA is the phytohormone most often cited for encouraging the breaking of seed dormancy. As mentioned above, H_2_O_2_ and other ROS can trigger the synthesis of GA. However, H_2_O_2_ is also potentially implicated in the regulation of ABA—a hormone that prolongs dormancy. Specifically, ABI1 and ABI2, both protein phosphatase 2Cs, exert negative control on ABA. These enzymes are reversibly inhibited by H_2_O_2_ in Arabidopsis, suggesting that H_2_O_2_ may indeed promote ABA signaling by inactivating negative regulators [47,48]. This seems to be a small point of controversy in the literature, as several citations seem to reach the opposite conclusion to what was intended by Meinhard et al. (2002) [48]. Barba-Espin and colleagues (2010) [41], for example, cite that “Treatment of dormant seeds with H_2_O_2_ results in a decrease in ABA levels. Moreover, H_2_O_2_ has been shown to inactivate the type 2C protein phosphatases ABI1 and ABI2, two enzymes involved in ABA signaling”. While the interpretation here is not specific, the inclusion of ‘moreover’ implies it aligns with the previous thought (e.g., that H_2_O_2_ decreases ABA levels). This observation highlights the need for follow-up work in this field and demonstrates the complex crosstalk that has been shown to occur between a variety of ROS and phytohormones [49]. 

Apoplastic •OH production increases in the radicle and the endosperm cap of imbibed *Lepidum sativum L*. and *Arabidopsis thaliana* (*L*.) *Heynh*. seeds prior to endosperm cap weakening and rupture [50,51]. This tissue weakening is critical for endospermic seed plants as the radicle requires both the seed coat and the endosperm to rupture before it can emerge. This result is consistent with earlier work in radish (*Raphanus sativus L*.) that not only demonstrated a distinct rise in •OH during germination, but also demonstrated that the exogenous application of •OH scavengers was sufficient to prevent germination [52]. Consistent with this mechanism, it has also been shown that apoplastic •OH is synthesized by plant cells in the absence of any exogenous reductants [53]. Further, juvenile, unstressed cells in the growing zone of maize roots generate ROS (including •OH) at the site of elongation, providing further support for the critical links between ROS production and root elongation [54]. ROS continue to be important to the developing root. It has been shown that during the final stages of seedling development, O_2_^•−^ increases at the same time the radicle elongates [55]. It has also been demonstrated that ROS are critical for the positive gravitropic response in maize seedlings. In this work, the inactivation of PtdIns 3-kinase was shown to significantly impair the typical gravitropic response of the roots. ROS production was experimentally blocked by a pretreatment of LY294002 and the prevention of ROS synthesis resulted in an ~50% reduction of gravitropism as estimated by root curvature [56]. 

In the mitotic root tips of both *Triticum turgidum* and *Arabidopsis thaliana*, it has been demonstrated that ROS levels must be maintained in homeostasis for proper mitotic microtubule system function. Elevated or insufficient ROS concentrations result in several detrimental phenotypes, including: the disappearance of microtubules, the inhibition of preprophase band formation, the delay of nuclear envelope breakdown at prometaphase, the prevention of perinuclear tubulin polymer assembly in prophase, and the loss of bipolarity of spindles during prophase, metaphase, and anaphase. Further, macrotubule formation was observed in cells with low ROS levels, and tubulin paracrystals were present in cells experiencing oxidative stress [57]. 

Taken together, a rich body of work now strongly supports the idea of an ‘oxidative window’ for successful seed germination. ROS are critical for signaling, hormone regulation, and the weakening of multiple structures of the seed, but ROS above a critical threshold can lead to cell damage and death [58].

### 2.2. Shoot and Flower Development

Along with the root apical meristem (RAM), the shoot apical meristem (SAM) is responsible for the continued growth and organ development in plants (Figure 1D). Control of the SAM is a complex process that involves a feedback signal between the WUSCHEL (WUS) homeobox protein and CLAVATA (CLV) peptides and receptors (reviewed by Clark 2001 [59]). This feedback system can be manipulated by a host of endogenous factors, including ROS (see Sankaranarayanan et al. 2020 for a detailed review [60]). It is becoming increasingly clear that ROS distribution dictates the boundary between cell division and cell differentiation in both shoots and roots. In the shoot, O^2•−^ and H_2_O_2_ accumulation maintains WUS and CLV3, or CLAVATA ligand 3 [61], and these ROS are established in a gradient as controlled by a set of peroxidases, as described in roots [9,62]. While O^2•−^ has been shown to be essential for stem cell maintenance, high levels of O^2•−^ are difficult to maintain as superoxide radical is typically catalyzed by superoxide dismutases (SODs) into H_2_O_2_ rather quickly. Expression analyses suggest that SODs are downregulated in plant stem cells, explaining the relatively high levels of O^2•-^ in this tissue. On the other hand, SODs are upregulated in the peripheral zone, encouraging cell differentiation [61]. WUS is generated in the rib meristem and diffuses into neighboring cells, forming a concentration gradient [63,64]. WUS represses cell differentiation and maintains the undifferentiated apical meristems [65]. Cells with low levels of WUS enhance CLV3, and alternatively represses CLV3 when WUS levels are high due to WUS competitively binding to the same cis-regulatory module as the CLV3 promoter [66]. CLV3, in the absence of high levels of WUS, then encourage stem cells to take on their ultimate cell fate, initiating organ development [67]. CLV3 then represses WUS expression in a regulatory loop [68]. As discussed above, this regulatory loop is ultimately controlled by ROS with O^2•−^ promoting WUS activity and stem cell maintenance and H_2_O_2_ promoting CLV activity and promoting cell differentiation and organ development. 

Adult plants lacking the ATP-dependent mitochondrial protease (AtFTSH4) exhibit premature SAM termination as they accumulate H_2_O_2_, producing internal oxidative stress [69]. Two more mutants, *msl2* and *msl3*, present with constituent osmotic stress, large, deform plastids and a shoot apex covered by callous tissue. The callous tissue itself relies on the upregulation of cytokinin (CK) receptors, the downregulation of cytokinin inhibitors, and the induction of WUS in a CK/WUS feedback loop [70]. The over production of plastid ROS is linked to increased CK production [71]. Plant regeneration from callus tissue in strawberry showed that H_2_O_2_ may well serve as a key signaling molecule in the process of bud primordium formation. H_2_O_2_ production is coincident with the emergence of meristematic function in the callus with exogenous H_2_O_2_ slightly promoting this function and DDC (N,N-diethyldithiocarbonate—an H_2_O_2_ generation inhibitor) decreased regeneration percentage [72]. Specific levels of H_2_O_2_ are required for proper leaf elongation, as demonstrated in maize [72]. 

ROS and nitric oxide (NO) levels vary greatly at different reproductive time points and in different floral organs [73,74]. In general, the stigmatic surfaces of angiosperm carpels are known to be high in ROS, specifically H_2_O_2_. More specifically, in *Arabidopsis thaliana* and *Senecio squalidus L*., the stigmatic papillae accumulate H_2_O_2_. H_2_O_2_ levels then fall once pollen grains adhere to the papillae [75]. Pollen grains are known to be relatively high in NO content, and NO has recently been implicated in pollen tube growth and development [76]. NO has been further implicated in ‘crosstalk’ with the ROS in a receptive stigma and may actually be important in initial pollen–stigma recognition [75], including the generation of self-incompatibility responses [60,77]—although more work needs to be done in this area. On pollen tube growth specifically, in *Nicotiana tobacum L*., it was discovered that NOX enzyme derived ROS are high in the growing tip of the pollen tubes. Further, a NOX inhibitor (diphenylene iodonium chloride [DPI]) and ROS scavengers successfully inhibit pollen tube growth in culture [78]. Pollen tubes navigate their way through the style of the carpel, enter the micropyle of an ovule, and then rupture at the site of the female gametophyte. The pollen tube does not rupture at various other high ROS locations, indicating a more sophisticated mechanism of sperm release. It has been shown both in vitro and in vivo that •OH are the most abundant ROS in the pollen tube tip and that they induce tube rupture in a process that requires Ca^2+^ and Ca^2+^ channel activation [79]. While there is a robust body of literature on pollen-derived ROS, it is also evident that the female gametophyte generates ROS via respiratory burst oxidase homolog (RBOH) activity, which also likely plays a role in pollen tube rupture, perhaps through cell wall weakening [80]. It has been known for some time that cytoplasmic Ca^2+^ is important for pollen tube growth, and that the filiform apparatus of the synergid cells are high in Ca^2+^ [81], with Ca^2+^ levels in the synergids reaching their peak with pollen tube rupture [82]. This, once again, creates a novel link between known physiological processes and their interactions or dependencies on ROS signaling. 

The mature female gametophyte, or egg sac, in angiosperm is commonly composed of seven cells: an egg and two synergid cells at the micropylar end, a diploid (or greater) central cell, and three antipodal cells located at chalazal end. In the mature female gametophyte, mitochondrial O^2•−^ and O_2_^2−^ are detected only in the central cell. During normal female gametophyte development, MSD1 (manganese superoxide dismutase 1) is expressed in high levels across the entire gametophyte, eventually becoming restricted to the egg and the synergid cells upon gametophyte maturity [83]. In fact, the *oiwa* mutant provides some evidence that MSD1 expression in the female gametophyte is essential to determining the fate of the central cell. In *oiwa* mutants, the high ROS levels typically only seen in the mature gametophyte’s central cell propagate to the egg and synergic cells and as a result, these cells take on the gene expression profile typical of the central cell [83]. It is typically only after pollination that the egg apparatus generates greater levels of ROS. The recurring theme of ROS homeostasis rears its head again here, as a mutant, athemn1, which is deficient in tetrapyrrole biosynthesis, shows increased ROS synthesis in both the male and female angiosperm gametophytes. This increase in ROS leads to non-viable pollen and a deformed embryo in which the polar nuclei do not fuse into a central cell [84], and in fact, several key mitochondrial ROS genes are active as early as the megasporocyte, or megaspore mother cell. Post fertilization, ROS are scrubbed from the female gametophyte. Mutants that are not able to arrest or scrub ROS show an arrest of embryo development [83].

## 3. How Plants Cope with Stress: Involvement(s) of ROS

### 3.1. Plant Responses to Common Abiotic Stressors

Drought stress and stress caused by high soil salinity are two of the most common abiotic plant stressors. Drought stress and increased soil salinity enhance ROS production and increase the incident of ROS-related damage [85,86]. This damage is exacerbated when combined with high light intensity [87,88]. These stressors disrupt photosynthesis and instead increase the rate of photorespiration, causing the production of ROS above homeostatic levels [89]. When plants receive excess light—more light than they can process via the light dependent reactions of photosynthesis—they rely on a variety of mechanisms to help prevent photodamage. These responses range from chloroplast avoidance movement, the movement of chloroplasts from the surface of the cells to the sides [90], acclimation through the modification of photosystem stoichiometry and antenna structures [91], to complex signaling pathways. When exposed to high light conditions, or rapidly changing light intensities, the change in redox state of the plastoquinone pool are coincident with the expression of genes associated with defense against oxidative damage [92,93]. In the mitochondria, the overall reduction level of the mitochondrial ubiquinone pool is the main driver of overall mitochondrial reactive oxygen output [94]. Of course, the rate at which mitochondria produce ROS is highly context-dependent, but the mitochondria are the primary source of ROS in non-photosynthetic tissue, including otherwise photosynthetic tissues in the absence of light [95]. 

Although several signaling pathways link drought and salt stress [96,97,98], the ABA signaling pathway in plants [99] has been shown to explicitly connect ROS and stress responsiveness. This pathway also requires the second messenger of cytosolic Ca^2+^, which increases sharply before stomatal closure [100]. Two guard cell–expressed NADPH oxidases, plasma membrane complexes, in the *Arabidopsis* genome are responsible for ABA-induced ROS synthesis and the chain of events that lead to the closure of the stomata. AtrbohD and AtrbohF, catalytic subunits of NADPH oxidases, function in the signaling pathway that mediates ABA activation of plasma membrane Ica channels, suggesting that ROS synthesis is the rate limiting step of abscisic acid signaling [101]. Ica channels have been shown to be stimulated by ROS [102], and ABA-insensitive mutants impair ROS activation of Ica channels, linking Ica channels to ABA signaling via ROS [103]. Further, ABA-induced ROS production is known to be impaired in *atrbohD/F* double mutants, but the pathway can be rescued by applying exogenous H_2_O_2_. ABA-activated SnRK2s also phosphorylate the plasma membrane NADPH oxidase RbohF, which when phosphorylated generates O^2•−^ in the apoplastic space. The O^2•−^ subsequently forms H_2_O_2_, a signaling molecule that mediates various ABA responses including stomatal closure [104]. ABA-induced H_2_O_2_ accumulation was first described in the guard cells of *Arabidopsis thaliana* [102] and *Vicia faba*, where it was discovered that H_2_O_2_ inhibited induced closure of stomata [105]. Drought stress causes a sharp increase in apoplastic ROS levels, which is required for the closing of the stomata by guard cells [106]. Therefore, ROS regulation links the critical function(s) of plants (e.g., photosynthesis and carbon capture) to their fates in the face of dominant abiotic stressors. 

### 3.2. Plant Responses to Biotic Stressors

It is now known that plants generate a burst of ROS in response to infection by virulent or even avirulent bacteria, fungi, and viruses [107,108]. Several mechanisms exist by which plants may generate oxidative bursts. While the nuances of when specific ROS generation mechanisms are active are still being elucidated, it is generally thought that protoplastic sources of ROS are most often linked with abiotic stressors [109,110], while membrane-bound NADPH oxidase, is associated with biotic stress [111]. NADPH oxidase produces bursts of superoxide anion in the apoplast, which can then be converted into H_2_O_2_ by superoxide dismutase. ROS synthesis in response to pathogens is biphasic: It begins with a low-amplitude, transient initial phase and is followed by a prolonged phase with increased magnitude conferring disease resistance [111]). Pathogens that escape recognition by the host fail to induce the second, higher magnitude wave and are thus unable to mount a defense against the pathogen, suggesting a critical link between the generation of ROS and the mounting of an effective immune response. Wheat cultivars exposed to a fungal pathogen, *Septoria tritici*, vary in their response to the pathogen. A resistant cultivar (cv. Stakado), which mounts a robust immune response, generates the previously described second, higher magnitude ROS burst. A second cultivar (cv. Sevin) is susceptible to the pathogen and fails to generate the second phase of the biphasic ROS response. In this system, even the second burst of H_2_O_2_ doesn’t not elicit a hypersensitive response in wheat plants, perhaps because this fungal pathogen is limited to growing through the apoplast [112]. Further, wheat leaves infiltrated with catalase scrubbed the leaf tissue of H_2_O_2_, increasing fungal penetration, colonization, and overall fungal biomass. This work suggests that H_2_O_2_ is not simply coincident with pathogen infection but is in fact critical to host defense [113]. These results are consistent with results from the study of fungal pathogens infecting barley leaves [114] and transgenic studies in potato wherein the insertion of a fungal gene encoding glucose oxidase–conferred resistance to bacterial soft rot disease and potato late blight [115]. H_2_O_2_ is also associated with the early events leading to the biosynthesis of phytoalexin [116].

ROS may be critical for establishing the hypersensitivity response (HR) of plants following infection and pathogen recognition [117,118]. Plants trigger an HR, which resultingly limits pathogen spread by initiating cell death at infection sites. Respiratory burst oxidase homolog (Rboh) genes are transcriptionally upregulated by pathogenic infections [119,120,121]. For example, elicitins, low molecular weight proteins secreted by *Phytophthora* [122] induce an HR in many plant species [123,124,125]. Genetic evidence for the function of Rboh in the pathogen-induced oxidative burst came from analyzing Rboh mutants and antisense lines. Here, the NADPH oxidase AtrbohF was shown to be important in the regulation of the hypersensitivity response [101,126]. In tobacco, after elicitation with cryptogein, tobacco cells transformed with antisense constructs of NtrbohD showed the same extracellular alkalinization as control plants, but they no longer produced ROS [121]. In the tobacco relative *Nicotiana benthamiana Domin*, the silencing of two rboh cDNAs, NtrbohA and NtrbohB, lead to lower levels of ROS production and consequently lower resistance to *Phytophthora infestans*. This work demonstrated that NtrbohA was expressed at low levels constitutively and transcripts were upregulated after leaf infiltration, whereas NtrbohB was induced by the protein elicitor INF1 from the pathogen. Both genes were shown to be critical for H_2_O_2_ accumulation and for resistance to *Phytophthora* [127]. Keeping with the theme that the dose makes the poison, in *Lycopersicon esculentum Mill*., infection by *Botrytis cinerea Pers*. alters the action of the plant peroxisomal antioxidant system, causing plant-generated ROS to damage plant tissue and enhance the speed of pathogen-induced tissue senescence [128]. 

The synthesis, scavenging, and signaling involved with ROS in plants is a rich field with relatively recent origins. In particular relation to ROS and biotic interactions, continuing work on the synthesis, compartmentalization, and function of these molecules is likely to unveil novel insights into the spatiotemporal interactions that shape plant development.

## 4. Abiotic and Biotic Interactions: Stress and Microbiome Structure

### 4.1. Links between Abiotic-Biotic Stress

ROS can be linked to both abiotic and biotic plant stress responses (see Jalmi and Sinha 2015 [129] for a comprehensive review; also see Figure 1 for an overview). It remains mechanistically unclear how ROS can connect abiotic and biotic stress responses and homeostasis in plants, but efforts to link these three factors have begun to shed light on the topic. For instance, Sewelam et al. (2019) uncovered that the *Arabidopsis* HSP17.4CI gene, a cytosolic class I small heat shock protein, is upregulated during abiotic (i.e., cold, drought, heat, high-light, and salt) and biotic (biotrophic plant pathogens) stress. Oxidative stress conditions were also shown to link abiotic–biotic stress pathways and ROS to HSP17.4CI [130]. Similarly, tomato SlAIM1 RNA interference plants with reduced abscisic acid-induced myb1 (SlAIM1) gene expression were shown to have increased susceptibility to the necrotrophic fungus *Botrytis cinerea* and increased sensitivity to salt and oxidative stress [131], suggesting that SlAIM1 integrates plant responses to pathogens and abiotic stresses by modulating responses to ABA. Nevertheless, in these cases and among many others (see Porter et al. 2020 for a recent meta-analysis [132]), natural or ‘live’ soils (i.e., autoclaved soils) were not used in the experiments, which may limit the frequency at which these proposed mechanisms operate given the relative complexity within natural systems (e.g., plant microbiomes and terrestrial ecosystems). In sum, investigating the community composition of plant microbiomes under both abiotic and biotic stress conditions should facilitate refined predictions of how plants may regulate ROS and respond to environmental perturbations.

Mapping trait-based characterizations of plant-associated microbes (such as ROS scavenging factors) onto whole microbial communities can obscure estimates on the functional ecology of complex, adaptive systems such as plant microbiomes [133,134,135]. For example, competitor microbes have been shown to limit the stress mitigating efforts of neighboring microbial symbionts [136,137], and synergisms among microbes can alter microbial impacts on plant fitness [32,138]. Therefore, presumed microbial symbionts (e.g., those capable of enhancing nutrient availabilities or warding of pathogens) may be rendered ineffective in their symbioses depending on the local composition of microbiota, or presumed plant pathogens may not exert archetypal pathogenesis. How these indirect and/or higher-order interactions [133] contribute to plant responses across large spatial scales remains unclear, but the interplay between and among microbes can drastically affect plant responses to abiotic and biotic stressors [139]. In contrast, keystone taxa (e.g., mycorrhizal fungi) can account for the majority of variance within plant microbiomes (i.e., microbiome architecture across space and time) (see Dastogeer et al. 2020 for a recent review [140]), which could eliminate the need to saturate efforts to uncover most multipartite interactions that do not involve mycorrhizal fungi and/or dominant microbial organisms. Thus, caution should be applied when attempting to generalize microbial functions across varied environmental contexts, particularly if microbial traits have only been analyzed in singular, artificial environments.

### 4.2. ROS and Plant Microbiome Structure and Function

Strong evidence suggests that ROS shape plant immunity and microbiota homeostasis [141,142]. Reports have shown that both plant and microbial produced ROS can initiate plant immune responses [13,143,144,145] and coordinate the abundance and diversity of microbial populations [146,147] across space and time (Figure 2). ROS gradients within plant compartments (e.g., roots and shoots) have also been shown to bias the functional structure of microbial communities (i.e., selection for ROS scavenging strains), which could explain compositional trends within plant microbiomes [148,149]. Both commensal and pathogenic microbes can illicit plant host immune responses (and ROS generation) via peptide-receptor binding interactions such as PAMP-PRR (e.g., flg22-FLS2) [29,30,150,151,152,153], but reports suggest that relatively high levels of exogenous ROS production are common features of pathogenic microbes, whereas ROS scavenging (and/or low net ROS production) has been continually linked to beneficial microbes [154,155,156,157]. Nevertheless, the threshold concentrations of ROS that frame these categorically bipartite host–microbe relationships remain unclear but likely vary as a function of host genotype, soil type and texture, resident microbiota composition, and proximity to plant structures. Similarly, the ephemeral nature of ROS (particularly O^2•−^and •OH) presents methodological challenges for their detection and trajectory within complex systems [7], but concerted shifts in microbiota concentration likely vary as a function of these ROS gradients (Figure 2B). Thus, fine-tuning our understanding of ROS fluxes within diverse plant microbiomes should help identify general and specific mechanisms that undergird host-recognition, microbe-microbe interactions, and the functional ecology of these complex systems. 

Recent communications have begun to resolve how plants regulate microbial responses via ROS in plant microbiomes. A report from Pfeilmeir et al. (2021) demonstrated that a lack of plant NADPH oxidase RBOHD (respiratory burst oxidase homologue), which facilitates ROS production, ushered in the rise of opportunistic bacterial pathogens (*Xanthomonas* sp.) and generally altered phyllosphere and endosphere microbiota compositions [142]. However, whether a loss of RBOHD directly impacts the growth of individual bacteria or indirectly reconfigures microbe–microbe interactions remains unclear. In contrast, Song et al. (2021) found that rbohf *Arabidopsis* mutants select a reproducible root microbiome that is enriched in *Pseudomonas* species and could resultantly increase plant fitness in natural conditions [158]. Similarly, Colaianni et al. (2021) demonstrated that commensal plant-associated bacteria harbor diverse immune-evading flg22 epitopes during unstressed states (i.e., low ROS levels), whereas immune-activating flg22 epitopes become enriched during physiological stressed states (i.e., high ROS levels). Importantly, these findings suggest that co-evolutionary processes likely drive the communication between commensal bacteria and plants to the end of maintaining homeostasis and diversity within plant microbiome communities [152] Continued efforts to understand the spatiotemporal dynamics of ROS-mediated plant microbiome restructuring will likely decode the language that connects plants and microbes.

### 4.3. Do Bacteria and Fungi Help Drive Plant Homeostasis Differently?

Bacteria and fungi constitute the majority of microbial biomass within plant microbiomes, and several reports (see Trivedi et al. 2020 for a comprehensive review [14]) have shown how they can drive the homeostasis of their plant host(s). A recent meta-analysis conducted by Porter et al. (2020) revealed that bacteria and fungi mitigate plant stresses (both abiotic and biotic) in paradigmatic ways [132]. During unstressed conditions, bacteria tend to benefit their plant host more than fungi, whereas mycorrhizal fungi appear to mitigate plant stress more effectively than bacteria. However, these general trends are not entirely binary: some bacteria were shown to significantly reduce abiotic and biotic stresses, and some mycorrhizal fungi were unable to ameliorate plant stress. The mechanistic drivers of these divergent outcomes also remain unclear, but the composition of the microbial community and plant phylogenetic classifications are suggested to predict the benefit(s) that a plant can obtain from microbes [159,160,161]. Biotic complexity of the rhizosphere microbiome, however, may not always impact the microbial benefits provided to the plant [132,162], and plant phylogenetic divergences may allow similar compositions of bioinoculants to exhibit similar plant responses. Given that bacteria and fungi remain dominant members of plant microbiomes [163,164,165], factorial experiments are required to resolve how organisms in these two domains interact in the context of plant homeostasis. Importantly, determining if discrete ROS regulation mechanisms exist across fungal and bacterial species could shed light on the functional ecology of plant development. 

### 4.4. Priority Effects in Plant Microbiomes: A Key Consideration for Effectively Implementing Bioinoculants 

**Figure 3:**
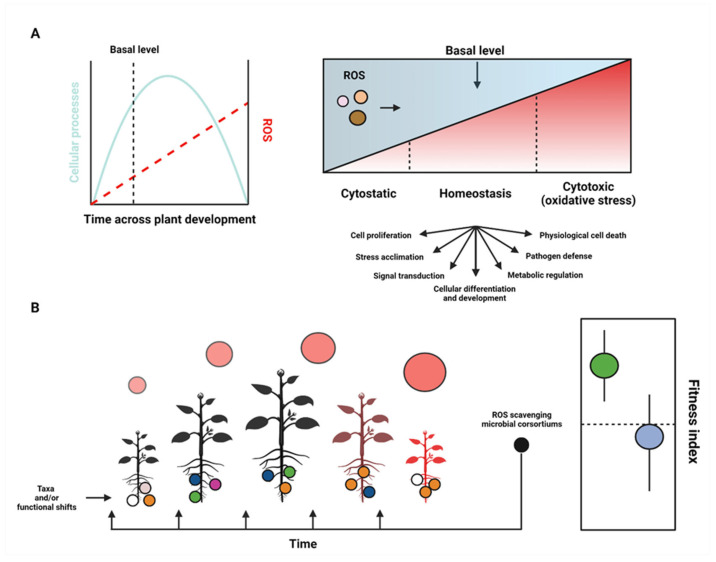
The ROS ‘oxidative window’ that balances plant growth, development, and senescence and integrates linkages between plant growth and microbiome composition. (**A**) ROS accumulation as a function of plant development, whereby the trajectory of plant development (i.e., lifespan) predictably trends along the tract of an arch, and ROS accumulate highest toward plant senescence. The balance of ROS, however, propels several homeostatic and developmental processes (see gradient plot above). (**B**) Given that microbial compositions shift along spatiotemporal scales of plant development and relative ROS concentrations can be tracked across plant development (circles above plant icons represent relative ROS concentrations, whereby circle size is proportional to ROS concentration), it follows that bioinoculants with antioxidant properties (i.e., ROS scavengers) should be applied at times and functional concentrations that optimize the intended outcomes of the plant or crop system (e.g., enhanced plant biomass, pathogen defense, delayed onset growth). For instance, microbes that exhibit strong antioxidant properties may facilitate cytostatic states, which could ultimately decrease plant fitness (see fitness index). Experiments geared to tackle the effects of bioinoculants equipped with antioxidative properties should gauge the spatiotemporal effects of the microbial community composition in addition to the effects on plant growth and development (yielding a fitness index that links microbial diversity and richness and plant health). For example, a hypothetical fitness index could encompass in planta ROS levels, microbiota composition, plant biomass, and time till senescence. To this end, resolving when ROS-scavenging bioinoculants exert their optimal effect(s) on plant growth and development can be achieved. Adopting such parameterizations will lead to a clearer understanding of how bioinoculants alter community dynamics and will help us better predict how unfavorable shifts in plant and microbial diversity can be reconfigured to buffer agroecological systems.

The timing of species arrival (i.e., priority effects) can affect the trajectory of microbe–microbe interactions and subsequently the collective interactions that manifest among microbiota and their plant host(s). As such, the degree to which microbes, plants, and ROS are linked depends on the temporal dynamics of both the interacting species and the temporal shifts of ROS (see Figure 3 for a general schematic), which resultantly can alter the impact that a focal species may have in terms of mitigating plant stress (e.g., bioinoculants) in natural systems. From niche pre-emption, whereby the early-arriving species depletes available resources for late-arriving species and limits the niche establishment for the late comer, to niche facilitation (i.e., established species enhance the establishment of late comer species) and niche inhibition (i.e., spatially-associated competition among species that is independent of nutrient limiting factors), the success of microbial plant symbionts depends, in part, on the spatiotemporal dynamics of the plant microbiota [32]. For example, fungal species have been shown to enhance the dispersal ability of associated bacterial species [139,166]), while other fungal species, depending on the microbial developmental stage, have been shown to both promote and deter bacterial growth [167]. Therefore, effectively implementing microbial plant stimulants (i.e., bioinoculants that facilitate plant growth and development) requires unpacking the effect that focal species have on the structure of the native plant microbiota across space and time. Addressing these questions will refine our ability to predict how selected microbes can sustainably be applied to mitigate crop stress and ultimately bolster crop production. Moreover, microbes that have been reported to regulate ROS and thus circumvent plant stress [168,169,170,171] should be examined through the lens of priority effects to holistically gauge their perceived microbe-mediated plant benefits—particularly since the spatiotemporal regulation of ROS must remain balanced. 

### 4.5. Leveraging Microbiota to Circumvent Plant Abiotic Stressors: A Key for Unlocking Plant Microbiome Functions across Space and Time

Abiotic stressors are known to alter plant physiology, development, succession, plant–soil feedbacks, and facilitation [172,173,174], and shifts in the abundance and composition of plant-associated microbiota have been documented repeatedly [175,176,177]. Numerous reports have begun to demonstrate that select microbiota may reduce toxic ROS levels [21,178,179,180,181] and effectively buffer abiotic stress for their plant host(s). For instance, Singh et al. (2020) found that *Pseudomonas fluorescens* could minimize ROS concentrations in rice plants under drought stress and subsequently bolster plant biomass [34]. Similarly, Tiepo et al. (2020) showed that *Azospirillum brasilense* and *Bacillus* species could enhance the level of enzymatic (ascorbate peroxidase and superoxide dismutase) and non-enzymatic (chlorogenic acid, gallic acid, rutin, and synapic acid) compounds in the seedlings of two Neotropical tree species (*Cecropia pachystachya* and *Cariniana estrellensis*) to the end of mitigating drought stress [157]. Others have shown similar findings in *Brassica napus* L., where various bacterial strains were shown to increase their host’s antioxidant production during heavy metal stress [182]. Nevertheless, lab-to-field hurdles remain in place for many of these bioinoculants. Thus, uncovering bioinoculants that mitigate abiotic plant stress in realistic conditions and effectively using them in agroecological systems [183,184,185,186] will undoubtedly require resolving their microbial ecology. 

Research on plant–plant interactions (PPIs) has started to unpack how interconnected distinct plants and their microbiota can be in natural systems (see Fahrig et al. 2011 and Mony et al. 2020 for complete reviews [187,188]). For instance, plants can function as sentinels by warning their neighboring plant of imminent stresses, where the ‘weaker’ plant adopts a similar composition of microbiota as the ‘stronger’ plant partner [189]. Moreover, volatile organic compounds (VOCs) have been reported to function as PPI and plant–microbe signaling cues [190,191], leading some to postulate that VOCs act as a common language among microbes and plants (see Deveau et al. 2018 for a comprehensive review [139]). VOCs have also been shown to be exploited by parasitic plants [192,193], and these parasitic plants have been shown to prefer their host’s repertoire of VOCs over those of non-host plant species [194]. However, it remains unclear how these PPIs affect the structure and function of native microbiota in real-time and even more unclear as to how multipartite interactions contribute to the regulation of plant ROS levels. Given that VOCs play vital roles in the regulation of ROS [195] and subsequently how microbe–microbe–plant interactions occur across space and time, ROS may function as an ‘operational signal’ to gauge the community dynamics within plant microbiomes (see Figure 3). Incorporating the multifaceted interactions that occur within plant microbiomes into current Earth system models remains challenging [196,197,198,199], but efforts to do so will lead to critical, predictive insights that (1) clarify how plants and microbes impact the evolutionary trajectories of one another and (2) answer critical questions related to plant–soil feedbacks. 

Several reports have linked plant–plant and plant–microbe interactions to overall crop productivity, and the use of microbial consortiums to bolster crop yields and obtain sustainable agricultural goals has been implemented regularly across many global regions [200,201]. For instance, Wagg et al. (2011) demonstrated that fungal identity and diversity relax plant–plant competition, which could significantly enhance plant productivity [202], whereas Song et al. (2021) recently demonstrated that mycorrhizosphere bacteria and plant–plant interactions could facilitate phosphorus acquisition in an intercropping agricultural system [158]. Similarly, Saia et al. (2020) showed that bacteria and AMF differentially benefit tomato and corn depending on the type of phosphorus that was present [203], and Qiao et al. (2017) reported that AMF can enhance crop biomass while suppressing weed biomass in intercropping systems [204]. However, understanding the mechanisms that drive multipartite interactions (e.g., plant–plant, plant–microbe, microbe–microbe) within agricultural systems remains challenging. New approaches will undoubtedly be required to overcome these challenges. For instance, Giraldo et al. (2019) proposed implementing nanomaterials to develop plant sensors that would allow monitoring and optimizing plant productivity, resource use, plant–plant signaling, and perhaps even plant–microbe interactions [205]. Although tremendous strides have been made over the last few decades in the field of microbial ecology, efforts geared toward elucidating the mechanisms that govern microbe–microbe and plant–microbe interactions will enable a robust understanding of how plants develop in complex environments. As such, novel approaches will facilitate our understanding of how plants develop in adaptive, complex environments, which requires understanding how their associated microbes interact with one another.

## 5. Conclusions and Future Perspectives

Plants and their associated microbiota function as adaptive, complex systems that continuously integrate environmental information to the end of obtaining homeostasis, and ROS signaling interconnects the abiotic and biotic stress responses of plants to their microbial constituents. Holistic approaches that investigate how ROS-linked abiotic–biotic feedbacks occur in nature will continue to develop our understanding of how these complex systems will function in the projected climatic regimes of the future, which will equip us with the knowledge to both preserve extant flora and fauna and engineer resilient plants for subsequent generations. 

## Data Availability

Not applicable.
